# Gut-Derived Metabolic Imbalance in Autism Spectrum Disorder: Toward the Concept of a Metabolic Subtype

**DOI:** 10.3390/nu18091442

**Published:** 2026-04-30

**Authors:** Ju Young Son, Yeyun Do, Jaemin Seo, Jeonghyun Choi

**Affiliations:** 1 Department of Rehabilitation Engineering, General Graduate School, Daegu Haany University, Gyeongsan 38610, Republic of Korea; 7057452@dhu.ac.kr (J.Y.S.); 7060164@dhu.ac.kr (Y.D.); seojaemin0319@dhu.ac.kr (J.S.); 2 Division of Rehabilitation Therapy, Adventure College, Daegu Haany University, Gyeongsan 38610, Republic of Korea; 3 Advanced Medical Device Research Center, Daegu Haany University, Gyeongsan 38610, Republic of Korea

**Keywords:** autism spectrum disorder, gut–brain axis, microbiome, short-chain fatty acids, propionate

## Abstract

Autism spectrum disorder (ASD) is highly heterogeneous in symptom onset and severity, comorbidities, and treatment responsiveness, challenging the notion of a single pathogenic mechanism. Increasing evidence indicates that some individuals with ASD exhibit prominent peripheral physiological alterations, including gastrointestinal (GI) dysfunction, gut microbial dysbiosis, immune imbalance, oxidative stress, and mitochondrial/energy metabolic vulnerability. In this context, gut-derived metabolites—particularly short-chain fatty acids (SCFAs)—have emerged as plausible modulators of the neurodevelopmental milieu through the expanded gut–immune–metabolic–brain axis. This review synthesizes: (i) SCFAs’ biogenesis and physiological roles, (ii) context- and developmental stage-dependent effects, (iii) the clinical heterogeneity of reported microbiome and SCFA alterations in ASD, and (iv) propionate as a frequently discussed candidate signal and the interpretive boundaries of preclinical evidence. Human studies show substantial inter-study variability in SCFA alterations (increases, decreases, or no differences), influenced by factors such as sample type (stool vs. blood), GI symptoms, diet, medication exposure, and analytical variability. Accordingly, SCFAs should not be treated as universal ASD biomarkers but rather as context-dependent metabolic signals relevant under specific clinical and biological conditions. Building on this premise, we propose the conceptual framework of “metabolic ASD” representing a metabolically informed dimension of biological variability in which peripheral metabolic–immune perturbations may contribute to neurodevelopmental vulnerability. To avoid premature causal claims, we outline design requirements for future research, including stratified study designs, longitudinal cohorts, and integrative multi-layer analyses. Ultimately, metabolic ASD should be positioned as a testable precision medicine research framework rather than a universal etiological model.

## 1. Introduction

### 1.1. Heterogeneity of Autism Spectrum Disorder and the Rationale for a Metabolic Subtype

Autism spectrum disorder (ASD) is a prototypical neurodevelopmental condition characterized by deficits in social interaction and communication, accompanied by restricted and repetitive patterns of behavior [1,2,3]. Despite its unified diagnostic framework, ASD exhibits marked interindividual variability across multiple dimensions, including symptom onset, severity, and treatment responsiveness, which represents a defining feature of the disorder.

Observational studies of infants and toddlers aged 18–24 months have demonstrated substantial variability in early communicative behaviors, while responses to early intensive behavioral interventions also show pronounced interindividual differences, with a considerable proportion of individuals failing to demonstrate significant improvement despite structured intervention [3,4,5,6]. Collectively, these findings underscore the limitations of conceptualizing ASD as a disorder driven by a single pathophysiological pathway and instead support the view of ASD as a multifactorial neurodevelopmental condition characterized by interacting biological dimensions.

This heterogeneity highlights the need for integrative conceptual frameworks that can account for the complex interactions among multiple factors shaping neurodevelopment. In this context, prior research has often focused on central nervous system (CNS) mechanisms, including synaptic dysfunction, altered neural connectivity, and disrupted neurodevelopmental signaling pathways.

Historically, research into ASD etiology has focused predominantly on genetic risk factors and disruptions in neurodevelopmental pathways. Variants affecting synaptic structure and function (e.g., SHANK3, NLGN3), chromatin remodeling (CHD8, ARID1B), transcriptional regulation (MECP2), and cell growth and signaling pathways (PTEN, TSC2), as well as alterations in mTOR and Wnt signaling, have been repeatedly implicated [7,8,9,10]. However, most individual risk genes account for less than 1% of ASD cases, and large-scale exome sequencing and meta-analytic studies have emphasized that many identified variants are rare. Consequently, genetic findings alone are insufficient to comprehensively explain the broad clinical and phenotypic heterogeneity observed across the ASD population. Recent advances in polygenic risk score approaches further support the notion that ASD arises from the cumulative effects of multiple genetic variants. Importantly, these models emphasize that genetic susceptibility operates in interaction with environmental and biological factors, rather than in isolation.

These limitations have prompted increasing recognition that, while genetic susceptibility provides an important foundation for ASD risk, additional biological layers—such as epigenetic regulation, environmental influences, and gene–environment interactions, as emphasized in recent ASD research frameworks—likely play important but not fully understood roles in shaping phenotypic expression. Within this context, peripheral metabolic environments may represent one such modulatory layer through which environmental influences interact with underlying genetic vulnerability. In parallel, animal studies have suggested that ASD-like phenotypes in preclinical models may be associated with peripheral physiological alterations, including gastrointestinal (GI) dysfunction and gut microbiota dysbiosis [11,12], immune dysregulation [13,14], and alterations in energy metabolism and mitochondrial function [15,16]. However, these findings are primarily derived from preclinical models, and their direct applicability to human ASD remains unclear.

Clinical studies further support this perspective, documenting a high prevalence of chronic GI symptoms and altered gut microbial composition [17,18], elevated systemic inflammatory markers [19], increased oxidative stress [20,21], and mitochondrial abnormalities [22,23], suggesting that peripheral biological environments may interact with neurodevelopmental vulnerability.

In light of this perspective, ASD can be more appropriately understood as a multifactorial neurodevelopmental condition shaped by interactions among genetic, environmental, and biological factors. Accordingly, this review adopts an integrative framework aimed at characterizing biological heterogeneity within the ASD population in terms of interacting and partially overlapping dimensions, without assuming discrete or mutually exclusive subgroup boundaries.

Within this context, we introduce the concept of “metabolic ASD” not as a discrete clinical entity or a biomarker-defined category, but as a hypothesis-generating conceptual framework. This framework refers to a potential dimension of biological variability in which peripheral metabolic alterations—including gut microbiota-derived metabolites, immune imbalance, and mitochondrial dysfunction—may function as a modifier of neurodevelopmental trajectories. Importantly, this framework does not assume direct causality between metabolic alterations and ASD, nor does it imply that such mechanisms are universally present across all individuals. Instead, it emphasizes context-dependent interactions between peripheral physiological states and neurodevelopmental vulnerability, with the aim of supporting biologically informed characterization of heterogeneity and guiding future investigations toward testable, context-specific mechanisms.

In this review, gut-derived metabolic imbalance is considered not as a primary causal mechanism or a definitive biomarker of ASD, but as a potential phenotypic modifier contributing to clinical heterogeneity. Accordingly, this work aims to provide a conceptual framework that integrates gut-derived metabolic signals into the broader pathophysiological landscape of ASD. Within this perspective, metabolic alterations are interpreted as interacting components that may contribute to variability across individuals, rather than defining discrete or metabolically distinct subgroups.

This framework supports a multidimensional approach that incorporates metabolic, immunological, and GI features as part of a context-dependent biological profile. From an operational perspective, this approach can be translated into testable research strategies by integrating these dimensions with clinical phenotyping.

### 1.2. The Expanded Concept of the Gut–Brain Axis and the Pathophysiological Significance of Gut-Derived Metabolites

The gut–brain axis is a conceptual framework describing bidirectional communication between the GI tract and the CNS, encompassing an integrated physiological network that links neural, immune, endocrine, and metabolic pathways [24]. Early models of the gut–brain axis primarily emphasized neural regulation mediated by the autonomic and the enteric nervous system. More recent evidence, however, has substantially broadened this concept by identifying gut microbiota and their metabolites as important modulators of gut–brain communication [24,25].

Within this host–microbe symbiosis, evidence across multiple experimental systems suggests that gut microorganisms generate a wide range of bioactive metabolites, some of which extend their influence beyond the local intestinal environment and reach distant organs via systemic circulation [26]. These microbiota-derived metabolites engage neural, immune, and metabolic pathways, forming an integrated signaling network linking peripheral physiological states to CNS function [27,28]. At the intestinal level, short-chain fatty acids (SCFAs) have been suggested to contribute to epithelial homeostasis and immune regulation, based largely on evidence from animal and in vitro studies, through receptor-mediated and epigenetic mechanisms [29,30,31,32]. SCFAs may influence enteric neural signaling by modulating neurotransmitter release through FFAR3 expressed on enteric nerve terminals [33].

Mechanistic studies, primarily in animal models and in vitro systems, suggest that intestinal dendritic cells and macrophages may respond to SCFAs via GPR109A signaling, promoting IL-10 production and facilitating immune tolerance through modulation of TLR4 signaling and induction of Foxp3^+^ regulatory T cells (Tregs) [34,35]. In parallel, tryptophan-derived metabolites such as indole-3-propionate have been shown, largely in preclinical studies, to activate aryl hydrocarbon receptor pathways, which may promote Tregs differentiation while suppressing Th17 polarization, thereby contributing to the attenuation of intestinal inflammatory responses [36,37]. However, these mechanistic insights are largely derived from preclinical models, and their direct relevance to human physiology remains to be fully established.

A subset of gut-derived metabolites can also access the CNS via the circulation and influence blood–brain barrier (BBB) integrity and CNS function through context-dependent mechanisms. For example, propionate has been reported to activate FFAR3 signaling in brain endothelial cells, as supported by evidence from human postmortem tissue and complementary experimental systems, thereby promoting NRF2-dependent antioxidant pathways while suppressing TLR4/CD14-mediated inflammatory signaling. Through these mechanisms, propionate has been associated with the restoration of tight junction protein expression, including claudin-5, occludin, and ZO-1, and with reduced BBB permeability [38,39]. From this perspective, the gut–brain axis extends beyond a purely neural conduit and is more appropriately viewed as an integrated regulatory system linking the gut, immune system, metabolic environment, and brain. However, despite the inclusion of human-derived data, much of the mechanistic understanding is based on experimental systems, and the direct applicability of these findings to human CNS function remains to be fully established.

Taken together, disruption of the intestinal barrier may permit microbial-derived metabolites to enter the systemic circulation and influence CNS-related processes through immune and metabolic pathways. Such processes involve periphery-to-brain signaling pathways and may, under certain conditions, be associated with neuroinflammatory responses, altered glial activity, and dysregulation of neuronal energy metabolism. In this context, emerging evidence suggests that microbiota-modulating dietary patterns, including the Mediterranean diet, may influence gut microbial composition and metabolic outputs such as SCFAs, although their clinical relevance in ASD remains uncertain.

This expanded view of the gut–brain axis provides a relevant theoretical framework for understanding neurodevelopmental disorders such as ASD. The developing nervous system is particularly sensitive to metabolic and immune signals, raising the possibility that subtle alterations in the gut environment during critical developmental windows, particularly during prenatal and early postnatal periods, may exert lasting effects on neurodevelopmental trajectories. Accordingly, gut microbiota and their metabolites have emerged as potential mediators linking environmental influences and host physiological states to ASD pathophysiology.

### 1.3. Research Trends on Short-Chain Fatty Acids and the Scope of This Review

SCFAs represent a major class of microbiota-derived metabolites mediating gut–brain communication and have been highlighted as important regulatory factors implicated in the gut microbial dysbiosis repeatedly reported in ASD [40]. SCFAs are primarily produced through the microbial fermentation of dietary fibers, with acetate, propionate, and butyrate constituting the principal components [41,42]. These metabolites are known to participate in the maintenance of intestinal homeostasis, modulation of immune responses, regulation of energy metabolism, and aspects of nervous system function [43,44,45,46]. Under physiological conditions, SCFAs generally act as homeostatic regulators that support host metabolic and immunological balance.

However, clinical studies in individuals with ASD have reported inconsistent alterations in SCFA concentrations, indicating that these physiological roles alone do not fully account for the observed heterogeneity. Such studies have also documented alterations in gut microbial composition accompanied by changes in SCFA concentrations and profiles; notably, the direction of these changes has been inconsistent, with reports of increased, decreased, or unchanged levels across studies [47,48]. This lack of concordance suggests that SCFA alterations are unlikely to represent a universal pathophysiological signature of ASD. Rather, they appear to reflect context-dependent phenomena shaped by multiple factors, including sample type, analytical methodology, age, dietary patterns, the presence of GI symptoms, exposure to medications or antibiotics, and overall systemic metabolic status.

Despite this heterogeneity, specific SCFAs have recurrently occupied a central position in ASD-related hypotheses, with propionic acid serving as a prominent example. In preclinical models, propionate has been associated with behavioral alterations, neuroimmune activation, mitochondrial dysfunction, and metabolic stress responses, leading to its frequent discussion in ASD-related metabolic research [1,49]. However, these findings are largely derived from experimental systems, and their relevance to human ASD remains to be established. In contrast, findings from human clinical studies have not consistently replicated these associations. Consequently, it is more appropriate to interpret propionate not as a singular causal agent of ASD, but as a context-dependent modifier that may influence neurobiological processes within metabolically vulnerable biological contexts.

Building on this perspective, the present review aims to reframe gut microbiota-associated metabolite imbalance, including SCFAs, within a heterogeneity-oriented conceptual framework, here referred to as “metabolic ASD.” To this end, we first provide a systematic overview of SCFAs’ biosynthesis, physiological functions, receptor-mediated signaling pathways, and their condition-dependent effects across developmental stages. On this theoretical foundation, subsequent sections focus on propionate as a representative case, critically examining both its relevance and its limitations in interpreting clinical heterogeneity and pathophysiological mechanisms in ASD.

## 2. Physiological Roles of Gut Microbiota and Short-Chain Fatty Acids

### 2.1. Composition of the Gut Microbiota and Mechanisms of Short-Chain Fatty Acid Production

The human gut microbiota is a complex microbial ecosystem composed predominantly of bacteria and plays a central role not only in digestion and nutrient absorption but also in immune regulation, metabolic homeostasis, and modulation of nervous system function. In healthy adults, the gut microbiota is typically dominated by the phyla Firmicutes, Bacteroidetes, Actinobacteria, and Proteobacteria, although their relative abundance and functional characteristics vary dynamically in response to diet, age, environmental factors, and medication exposure [50,51].

Gut microorganisms ferment dietary fibers and resistant carbohydrates that are otherwise indigestible by the host, generating a range of metabolites, among which SCFAs represent the most prominent end products. SCFAs are generally defined as fatty acids containing two to six carbon atoms, with acetate, propionate, and butyrate constituting the principal components. In the human colon, these SCFAs are typically present at an approximate molar ratio of 60:20:20. Under physiological conditions, total SCFA concentrations range from approximately 70–140 mM in the proximal colon and 20–70 mM in the distal colon. Both absolute concentrations and relative proportions of SCFAs are influenced by dietary composition, microbial metabolic activity, and host absorptive capacity, and are therefore often used as functional indicators of gut microbial activity [52,53].

SCFA production occurs through distinct metabolic pathways that depend on microbial species composition and community structure. Members of the phylum Bacteroidetes, particularly species within the genus Bacteroides, predominantly produce acetate and propionate. Propionate synthesis in these organisms commonly proceeds via the succinate pathway, mediated by enzymes such as methylmalonyl-CoA transcarboxylase [54]. In contrast, several taxa within the phylum Firmicutes, especially those belonging to the Clostridiales cluster (e.g., *Faecalibacterium prausnitzii*, *Roseburia intestinalis*, *Eubacterium hallii*, and members of the genus *Anaerostipes*), are major butyrate producers. These bacteria primarily generate butyrate through the acetyl-CoA pathway during carbohydrate fermentation. Notably, increased relative abundance of Firmicutes has been reported to correlate positively with colonic butyrate concentrations (r = 0.68 ± 0.21) [55].

Within the phylum Actinobacteria, species of the genus *Bifidobacterium* (including *B. longum* subsp. *infantis*, *B. bifidum*, and *B. breve*) predominantly produce acetate and lactate via the bifid shunt pathway, converting glucose into acetate and lactate at an approximate molar ratio of 3:2 [56]. These intermediate metabolites—particularly lactate, acetate, and succinate—can subsequently serve as substrates for other microbial taxa through cross-feeding mechanisms. For example, lactate and acetate are further metabolized into butyrate by specialized butyrate-producing bacteria such as *Anaerostipes caccae*, *Eubacterium hallii*, and *Anaerobutyricum soehngenii*, contributing to approximately 20% of total colonic butyrate production [57]. Such functional interactions among microbial taxa are key determinants of both the composition and diversity of the SCFA pool in the gut.

SCFAs produced within the intestinal lumen are predominantly absorbed and utilized locally by the colonic epithelium. More than 95% of colonic butyrate is consumed locally as a primary energy source for colonocytes, whereas approximately 64% of acetate and 91% of propionate are transported via the portal circulation to the liver. Upon hepatic uptake, propionate and butyrate are preferentially extracted, with an estimated 70–90% being metabolized before reaching the systemic circulation. In contrast, more than 90% of acetate is estimated to escape hepatic extraction and enter the peripheral circulation, where it can be utilized by extrahepatic tissues. Accordingly, the ultimate physiological impact of SCFAs reflects not only their intestinal production but also epithelial absorption, hepatic metabolism, and the host’s systemic metabolic state. These characteristics underscore the role of SCFAs as functional mediators that may link gut microbial activity to host physiology, rather than as simple byproducts of microbial fermentation [58,59].

### 2.2. Physiological Functions of Major Short-Chain Fatty Acids and Gut–Immune–Neural Homeostasis

Microbiota-derived SCFAs function not merely as energy substrates but as key physiological signaling molecules that broadly regulate the intestinal mucosal environment, immune responses, and neural function. Under normal physiological conditions, appropriate SCFA production—estimated at approximately 500–600 mmol/day, with a characteristic physiological distribution among acetate, propionate, and butyrate—provides an essential foundation for maintaining functional integration across the gut–immune–neural axis [60]. Evidence from experimental studies suggests that butyrate at physiological concentrations (~0.5 mM) may induce interleukin (IL)-22 production in intestinal CD4^+^ T cells and innate lymphoid cells via GPR41 signaling, while being associated with increased expression of tight junction-associated proteins, including claudins, occludin, and ZO-1, in intestinal epithelial cells. In parallel, maintenance of physiological cerebrospinal fluid concentrations of acetate (0–171 μM), propionate (0–6 μM), and butyrate (0–2.8 μM) has been associated with increased expression of claudin and occludin at the BBB, thereby potentially supporting BBB integrity, although these observations are largely derived from experimental systems [60,61]. Collectively, these observations suggest that SCFAs under physiological conditions may act as important regulators of gut–immune–neural homeostasis.

Despite belonging to the same metabolite class, individual SCFAs exert distinct biological effects within the gut–immune–neural axis due to differences in their sites of production, tissue distribution, metabolic fate, and receptor affinities. Accordingly, this section delineates the representative physiological roles of acetate, butyrate, and propionate under normal conditions.

Acetate is the most abundant SCFAs produced in the gut and exhibits relatively efficient transfer into the systemic circulation [58,60]. Under physiological conditions, approximately 64% of acetate in the portal circulation is extracted by the liver, while the remaining fraction reaches peripheral tissues. In the liver, acetate serves as a precursor for acetyl-CoA, contributing to fatty acid and cholesterol synthesis and modulating lipogenesis via AMP-activated protein kinase (AMPK) activation. In skeletal muscle, acetate functions as an energy substrate and has been reported to increase myoglobin and GLUT4 expression, thereby enhancing oxygen utilization and insulin sensitivity. In adipose tissue, acetate promotes the expression of genes associated with lipolysis and increases oxygen consumption, contributing to systemic energy homeostasis [58,62]. Extending these metabolic observations to central nervous system function, Frost et al. (2014) reported that acetate derived from dietary fiber fermentation crosses the BBB and may influence hypothalamic signaling pathways, based on findings from PET-CT and 13C high-resolution magic-angle spinning spectroscopy, with associated changes in neuropeptide expression and AMPK/ACC signaling linked to appetite regulation [63]. More recently, Forte et al. (2024) reported a mechanism by which acetate may suppress the excitability of hypothalamic orexin/hypocretin (OX/Hcrt) neurons via GPR43 signaling, further supporting the role of acetate in gut–brain metabolic communication [64].

Butyrate serves as the primary energy source for colonic epithelial cells, promoting ATP production through β-oxidation and the tricarboxylic acid (TCA) cycle. In studies using both germ-free and C57BL/6 mice, Donohoe et al. (2011) reported that butyrate deprivation in colonocytes from germ-free mice resulted in an approximately ~56% reduction in ATP levels, suggesting impaired cellular energy metabolism [65]. Physiological butyrate supplementation effectively reverses these metabolic deficits, thereby preserving epithelial integrity and function. In addition, butyrate supports intestinal barrier function and epithelial differentiation through metabolic and epigenetic regulation [43,66].

Mechanistic studies in animal models and in vitro systems suggest that butyrate and propionate may exert anti-inflammatory properties through GPR43-mediated signaling and HDAC inhibition, thereby potentially promoting differentiation of naïve CD4^+^ T cells into FoxP3^+^ Tregs. This process may be associated with increased expression of CD39 and PD-L1 and enhanced IL-10 production, and reduced production of pro-inflammatory cytokines such as TNF-α and IL-6. Collectively, these findings suggest that SCFAs may favor tolerogenic differentiation of dendritic cells and help maintain immune homeostasis [67,68].

Propionate generated through intestinal fermentation is transported to the liver, where portal concentrations increase by approximately 35- to 100-fold and hepatic propionyl-CoA levels rise by 8- to 18-fold. Within hepatocytes, propionate contributes to hepatic metabolism and systemic energy regulation, influencing gluconeogenesis and metabolic flux through integrated pathways [69,70]. In experimental studies using an ex vivo adipocyte system derived from rats, Heimann et al. (2014) reported that propionate and butyrate may suppress hormone-stimulated lipolysis, reduce de novo lipogenesis, and enhance insulin-stimulated glucose uptake, supporting a role for SCFAs in lipid metabolism and insulin sensitivity [71]. In line with these findings, Weitkunat et al. (2016) reported that propionate supplementation in a high-fat diet model reduced hepatic triglyceride content by approximately 40%, suppressed expression of sterol regulatory element-binding factor 1 and related lipogenic enzymes, and increased odd-chain fatty acid production, resulting in improved insulin sensitivity [72].

Within the immune system, propionate exhibits high affinity for GPR43 (FFAR2) and directly acts on FoxP3^+^ Tregs in the colonic lamina propria, increasing histone acetylation through inhibition of HDAC6 and HDAC9 and enhancing the expansion and function of IL-10–producing Tregs. Propionate also upregulates expression of the homing receptor GPR15, facilitating colonic localization of Tregs and supporting immune tolerance [73]. In addition, propionate has been suggested to influence CNS accessibility through circulation-dependent mechanisms, although these effects are likely context-dependent [38]. Notably, portal venous SCFA concentrations are approximately fivefold higher than those in peripheral circulation (375 μmol/L vs. 79 μmol/L), and despite relatively high hepatic clearance, propionate may be capable of achieving physiologically relevant BBB exposure, suggesting a potential role as a mediator within the gut–brain axis [63,74].

Collectively, major SCFAs exhibit distinct yet complementary metabolic and immunological properties and are thought to act in a tissue-specific manner from intestinal fermentation through systemic circulation to the CNS. A clear understanding of SCFA functions under physiological conditions therefore provides an essential reference framework for interpreting the significance of SCFA imbalance observed under pathological states.

### 2.3. SCFAs’ Signaling Mechanisms: Receptor-Mediated Pathways and Epigenetic Regulation

The physiological effects of SCFAs extend beyond their roles as energy sources or metabolic substrates and are mediated through both extracellular receptor-dependent signaling and intracellular epigenetic regulation. At the cellular level, SCFAs signal primarily via G protein-coupled receptors, including GPR41/FFAR3, GPR43/FFAR2, and GPR109A, as well as through receptor-independent inhibition of HDACs [45,75]. These complementary mechanisms enable both rapid cellular responses and longer-term modulation of gene expression across tissues.

Evidence from experimental studies suggests that SCFA-sensing receptors are expressed across intestinal and immune cell populations and may be involved in regulating immune responses, epithelial barrier integrity, and metabolic processes [76,77]. Through these pathways, SCFA signaling may contribute to coordinated regulation of metabolic and inflammatory responses both locally and systemically.

In parallel with receptor-mediated signaling, SCFAs have been reported to influence gene expression through inhibition of HDAC activity [31,32]. HDAC inhibition is associated with increased histone acetylation, thereby altering chromatin accessibility and enabling sustained transcriptional reprogramming. This epigenetic mode of action allows SCFAs to function not only as transient metabolic signals but also as potential modulators of longer-term cellular phenotype and function [78]. Experimental studies further demonstrate that SCFA-induced histone acetylation may lead to sustained transcriptional and functional cellular changes [79]. In immune and epithelial cells, these epigenetic mechanisms may contribute to the regulation of inflammatory responses and barrier function, highlighting the context-dependent nature of SCFA signaling [80,81].

Collectively, SCFA signaling reflects an integrated regulatory system shaped by concentration, exposure duration, and tissue-specific context, rather than a linear or uniform pathway. Such properties provide a framework for understanding how identical metabolites may exert divergent biological effects across physiological and pathological conditions.

### 2.4. Developmental Stage- and Context-Dependent Actions of SCFAs

The biological effects of SCFAs are maintained within specific concentration ranges—typically within physiological contexts at low millimolar levels (approximately 1–5 mM). Outside these ranges, particularly under high-concentration exposure (≥20 mM), the same metabolites can elicit qualitatively distinct or even opposing biological responses, reflecting a bidirectional dose–response relationship [82,83].

SCFAs therefore exhibit strong context-dependent behavior, with physiological and excessive exposures producing distinct biological effects influenced by concentrations, cellular environment, and inflammatory status. Under certain conditions, excessive or imbalanced SCFA exposure has been associated with activation of inflammatory pathways, including NLRP3 inflammasome signaling and increased production of pro-inflammatory cytokines, which may contribute to neuroinflammatory responses [84,85].

For clarity, the developmental windows discussed in this review can be broadly categorized into prenatal (maternal–fetal), early postnatal (infancy), and later developmental stages. These periods differ in terms of neurodevelopmental processes, microbiota establishment, and immune maturation, and may therefore confer distinct vulnerabilities to metabolic and inflammatory perturbations relevant to ASD.

In addition to concentration dependence, the physiological impact of SCFAs is strongly influenced by developmental stage. During fetal and early postnatal periods, when the nervous system undergoes rapid formation and remodeling, sensitivity to metabolic and immune signals is markedly heightened. This increased vulnerability reflects the presence of critical developmental windows characterized by elevated neural plasticity compared with adulthood [86]. During pregnancy, maternal gut microbiota and their derived metabolites, including SCFAs, have been reported to influence fetal neurodevelopment [87]. Similarly, early infancy (approximately 0–3 months of age) represents a period of rapid microbial colonization and increasing SCFA production, during which alterations in SCFA profiles may influence subsequent neurodevelopmental trajectories [88,89,90].

Key neurodevelopmental processes occurring from late gestation through early postnatal life—including myelination, synaptogenesis, and microglial maturation—have been shown, primarily in experimental studies, to be particularly sensitive to neuroinflammatory signaling and metabolic perturbations mediated by maternal inflammation and gut microbiota-derived SCFAs [91,92]. These findings suggest that SCFA exposure during early developmental periods may exert disproportionate influence on neural circuit formation relative to later life stages.

Clinical evidence further supports this developmental sensitivity. Alterations in maternal circulating SCFA concentrations during pregnancy have been associated with neurodevelopmental outcomes in early infancy. In a cohort study of 357 mother–infant pairs, lower maternal serum concentrations of acetate, butyrate, and isobutyrate during the first trimester were associated with significantly higher Bayley-III language and psychomotor development scores at 40 days of age compared with higher-exposure groups. In contrast, propionate exhibited a non-linear association, with optimal developmental, mood, and temperament scores observed within an intermediate concentration range [93]. These findings indicate that the developmental effects of SCFAs are not linear and support the existence of an optimal exposure window.

Accumulating evidence suggests that SCFA exposure patterns during these sensitive periods may influence long-term effects on gut–brain axis function, mitochondrial energy metabolism, neuroimmune interactions, and glial activation states [25,92]. This contrasts with the role of SCFAs in mature individuals, where they primarily function as metabolic substrates or physiological signaling molecules. During development, SCFAs may act as critical modulators of the neurodevelopmental milieu. Consequently, imbalances in SCFA exposure—either excess or deficiency—during fetal and neonatal stages have been proposed as potential modulators of neurobiological processes associated with neurodevelopmental disorders, including ASD [40,94,95].

Moreover, SCFA actions are tissue-specific. Identical SCFA signals can elicit divergent responses across intestinal epithelium, peripheral immune compartments, the BBB, and the CNS microenvironment [39,44,46,96]. Such tissue-specific responsiveness provides a physiological basis for how alterations in the gut metabolic environment may extend beyond local intestinal effects to potentially influence CNS function.

In summary, while SCFAs serve as essential mediators of gut–immune–neural homeostasis under physiological conditions, their biological effects are highly dependent on concentration, developmental timing, and tissue context. These condition-dependent properties provide a critical conceptual framework for interpreting gut microbiota and SCFA imbalances observed in individuals with ASD and help explain the lack of consistency across clinical findings.

## 3. Gut Microbiota and Short-Chain Fatty Acid Imbalance in Autism Spectrum Disorder

### 3.1. Alterations in Gut Microbiota Composition in Individuals with ASD and Their Functional Implications

Reported alterations in SCFA levels in individuals with ASD have been inconsistent across human studies, with both increases and decreases described for acetate, propionate, and butyrate depending on study design and cohort characteristics. Table 1 summarizes representative quantitative studies illustrating this heterogeneity across cohorts. This variability reflects differences in biological and methodological context, including sample source, GI symptom stratification, developmental stage, and analytical methodology, with no uniform direction of change observed across individual SCFAs.

As discussed in the preceding sections, the gut microbiota and its derived metabolites play important roles in maintaining functional integration across the gut–immune–neural axis under physiological conditions. Against this reference framework, compositional alterations in gut microbial communities repeatedly reported in individuals with ASD warrant consideration not merely as differences in microbial diversity, but as potential modulators of host metabolic and immune regulatory environments.

Across multiple case–control studies and systematic reviews, individuals with ASD have been reported to exhibit reduced alpha diversity alongside significant separation in beta diversity-based community structure compared with typically developing controls [99,100,101]. In a meta-analysis by Iglesias-Vázquez et al. (2020), children with ASD (*n* = 493) showed significantly higher relative abundances of Bacteroidetes (14.33% vs. 10.97%, *p* = 0.002), Firmicutes (13.42% vs. 10.77%, *p* < 0.001), and Proteobacteria (0.09% vs. 0.02%, *p* < 0.001) compared with controls (*n* = 404) [102]. The Bacteroidetes/Firmicutes ratio was modestly higher in ASD (0.69) than in controls (0.44), although the direction of this ratio varied substantially across studies [102]. In contrast, a large cohort study by Li et al. (2024), involving 957 individuals with ASD and 161 controls reported a reduced Bacteroidetes/Firmicutes ratio in the ASD group (0.54 vs. 0.89, *p* < 0.05), together with significant group separation in beta diversity analyses, with the first principal coordinate (PCO1) explaining 12.56% of total variance [99].

At the genus level, selective increases and decreases in specific taxa have been reported (Table 2). Reductions in fermentative genera such as *Prevotella* (adjusted *p* = 0.04), unclassified Veillonellaceae (adjusted *p* = 0.04), and *Coprococcus* (adjusted *p* = 0.06) have been relatively consistently observed across studies [103]. Conversely, meta-analytic data indicate significant enrichment of *Bacteroides* (ASD 9.04% vs. control 4.69%, *p* < 0.001) and *Clostridium* (ASD 0.74% vs. control 0.16%, *p* < 0.001) in individuals with ASD [102].

Alterations in taxa associated with SCFA production have also been reported, although with substantial inter-study variability. For example, *Faecalibacterium prausnitzii* was reported to be increased in ASD in a meta-analysis (6.84% vs. 5.00%, *p* = 0.040), whereas individual studies have documented both increases and decreases, reflecting limited consistency across cohorts. Such discrepancies are likely attributable to differences in age ranges, taxonomic resolution (genus vs. species), and analytical methodologies [102,104,105]. Similarly, *Bifidobacterium* abundance was reduced in meta-analytic comparisons (ASD 0.46% vs. control 0.89%, *p* < 0.001), yet individual studies again reported conflicting results, underscoring methodological and population-level heterogeneity [101,102,104,106].

Collectively, these alterations in SCFA-associated taxa have been interpreted as potential indirect evidence of a perturbed intestinal metabolic environment in ASD. However, most studies have relied on functional prediction approaches such as PICRUSt2, limiting the ability to robustly link compositional changes to measured metabolite profiles [107]. As a result, causal or quantitative relationships between microbial shifts and SCFA alterations remain insufficiently established.

Importantly, the overall pattern of gut microbiota alterations reported in ASD lacks consistency across studies, making it difficult to define a universal microbial signature characteristic of ASD. This variability likely reflects the influence of multiple confounding factors, including age, dietary habits, the presence or absence of GI symptoms, antibiotic exposure, and differences in 16S rRNA sequencing platforms and bioinformatic pipelines [100,103,108,109,110]. Notably, some studies have reported more pronounced microbial alterations in ASD subgroups with co-occurring GI symptoms. For instance, individuals with ASD and GI symptoms exhibited selective increases in Sutterella, Roseburia, and Fusobacterium, accompanied by markers of increased intestinal permeability (elevated zonulin: 20.28 ± 5.21 μg/g; reduced lysozyme: 799.86 ± 672.91 μg/g). In contrast, ASD individuals without GI symptoms (*n* = 11) showed increases in Klebsiella and Lactobacillus without corresponding changes in zonulin or lysozyme levels relative to controls [111]. However, Kang et al. (2013) reported that reduced microbial diversity correlated with ASD symptom severity but not with GI symptom severity, suggesting that dysbiosis may occur independently of overt GI manifestations [103].

Overall, while gut microbiota alterations are a recurrent finding in ASD, their directionality and magnitude remain highly heterogeneous, likely reflecting functional reorganization of microbial communities rather than a uniform taxonomic shift. In the absence of consistent metabolite-level validation and given the reliance on predictive functional analyses, these alterations are more appropriately interpreted as context-dependent modulators of metabolic and immune environments, rather than as a singular etiological driver of ASD. This framework provides an essential premise for interpreting clinical studies examining SCFA concentration changes in individuals with ASD, which are addressed in the following section. Taken together, these observations support the need for stratified analytical approaches in which metabolic and microbial features are interpreted in combination, rather than as isolated variables, to better capture biologically meaningful variability across individuals.

### 3.2. Clinical Heterogeneity of Reported Short-Chain Fatty Acid Alterations in Individuals with ASD

In parallel with reports of altered gut microbiota composition in individuals with ASD, numerous clinical studies have attempted to quantify changes in the intestinal metabolic environment by measuring SCFA concentrations in fecal samples or blood [59,97,98]. However, these studies have not yielded consistent patterns of increase or decrease for specific SCFAs, instead reflecting substantial inter-study variability and clinical heterogeneity [47,97,98]. As summarized in Table 1, no consistent directional pattern is observed across individual SCFAs. This variability reflects the combined influence of biological and methodological factors summarized in Table 3.

Reported SCFA alterations differ by metabolite. Several studies have documented increased fecal concentrations of specific SCFAs—most notably propionic acid—in some of individuals with ASD. For example, elevated fecal propionate levels have been reported in children with ASD accompanied by constipation compared with typically developing controls [97]. In contrast, Wang et al. (2019) found no significant differences between ASD and control groups in the relative proportions of ten measured SCFAs, including propionate (22.2% vs. 21.4%, *p* > 0.05) [47]. Other cohorts have reported opposing patterns, with reduced acetate and butyrate concentrations alongside increased valerate levels in ASD (acetate: 732.4 vs. 899.9 μmol/g; butyrate: 413.2 vs. 563.3 μmol/g; ASD < control) [48]. Similar inconsistencies have been reported for acetate and butyrate across studies, with findings ranging from increases to decreases or no detectable differences [47,98]. Taken together, these discordant results indicate that alterations in SCFA composition cannot be reliably defined as a universal biological feature of ASD [40].

One important contributor to this heterogeneity is the type of biological specimen used for SCFA measurement. Fecal SCFAs primarily reflect local concentrations within the colonic lumen, where microbial fermentation and utilization of dietary fibers and carbohydrates occur. In contrast, circulating SCFAs represent the integrated outcome of intestinal absorption, first-pass metabolism in the portal–hepatic system, and subsequent utilization or clearance by peripheral tissues [94,98]. Consistent with this distinction, metabolomic studies that simultaneously analyzed fecal and plasma samples from the same ASD cohorts have shown that metabolite panels and signal intensities that distinguish ASD from controls differ substantially between compartments, indicating that luminal and systemic measures capture distinct aspects of host–microbiome metabolism [112,113,114]. These findings underscore the importance of considering sample-specific physiological context when interpreting SCFA data.

Clinical heterogeneity within ASD populations further amplifies variability in reported SCFA profiles. Subgroups of individuals with ASD who present with GI symptoms, particularly constipation, have more frequently exhibited elevated fecal propionate and valerate concentrations compared with controls [97,98]. However, such patterns have not been consistently reproduced across cohorts differing in age range, definitions and assessments of GI symptoms, dietary composition, and exposure to medications or antibiotics [40,94]. This lack of reproducibility suggests that reported SCFA alterations are more likely to characterize specific clinical subgroups rather than the ASD population as a whole.

Methodological differences also pose notable challenges for cross-study comparison. SCFA quantification has been performed using diverse analytical platforms, including gas chromatography–flame ionization detection (GC–FID), gas chromatography–mass spectrometry (GC–MS), and liquid chromatography–tandem mass spectrometry (LC–MS/MS) [115,116,117]. Variations in sample storage conditions, extraction protocols, internal standards, and reporting units (e.g., μmol/g feces, μmol/mL plasma, or relative proportions) can yield substantially different quantitative outcomes even from comparable biological samples [112,115,118]. Moreover, many studies are limited by small sample sizes and cross-sectional designs, constraining statistical power and reproducibility [47,48,97].

In summary, although a substantial body of quantitative data on SCFA concentrations in ASD has accumulated, the direction and magnitude of reported alterations vary widely across studies, reflecting pronounced clinical heterogeneity. These findings indicate that individual SCFAs are unlikely to serve as universal pathophysiological markers of ASD and are better interpreted as context-dependent metabolic readouts shaped by clinical characteristics, gut microbiota composition, dietary and pharmacological exposures, and methodological factors. Accordingly, future research may benefit from moving beyond treating SCFAs as a homogeneous group and instead examining acetate, propionate, butyrate, and other SCFAs individually, with careful consideration of their distinct physiological, immunological, and neurobiological effects in relation to ASD. While Table 1 provides a structured summary of representative studies, additional reports further support the marked heterogeneity of SCFA alterations, although differences in study design, sample types, and reporting formats limit direct cross-study comparability.

### 3.3. Selective Associations Between Propionate Alterations and ASD Phenotypes

As discussed in the preceding sections, alterations in SCFAs observed in individuals with ASD are characterized by substantial clinical heterogeneity, limiting the ability to generalize findings from a single metabolite. Nevertheless, when examined individually, propionate has consistently emerged as a focal point of investigation.

The recurrent emphasis on propionate can be partly attributed to two main considerations. First, several clinical studies have reported alterations in fecal or circulating propionate levels in individuals with ASD, with such changes appearing more pronounced in subgroups presenting with co-occurring GI symptoms [97,98,119]. Second, preclinical studies have suggested that exogenous propionate exposure or experimental manipulation of propionate-related metabolic conditions may be associated with behavioral alterations, dysregulated neuroimmune responses, and disturbances in energy metabolism [1,120,121]. Collectively, these findings suggest that propionate may possess biological properties that enable interactions with neural and metabolic systems.

Importantly, however, these observations do not imply that propionate functions as a direct causal agent in ASD. In clinical contexts, associations between propionate levels and ASD-related phenotypes have not been consistently replicated across all cohorts and vary according to study design, biological specimen analyzed, and characteristics of the study population [40,47,48,97]. This variability suggests that the biological effects of propionate may not be universally operative across the ASD population, but instead manifest selectively under conditions of specific metabolic and immune vulnerability.

From this perspective, propionate is more appropriately conceptualized not as a definitive etiological marker of ASD, but as a candidate signaling molecule whose neurobiological impact may be modulated by broader metabolic and immunological contexts. Increases in propionate should therefore be interpreted not as an independent pathogenic factor, but as a context-dependent component interacting with gut microbial composition, intestinal barrier integrity, and systemic metabolic and immune states.

In summary, among the SCFA alterations reported in ASD, propionate has consistently attracted attention despite pronounced clinical heterogeneity. Its selective consideration serves not to generalize SCFA imbalance as a universal feature of ASD, but to provide a focused entry point for exploring mechanistic possibilities within biologically plausible subgroups. In this regard, propionate may provide a conceptual bridge toward subsequent discussions of neurobiological mechanisms within the conceptual framework of “metabolic ASD.” These findings support the need for stratified analytical approaches rather than uniform interpretation across individuals.

## 4. Neuro-Pathophysiological Implications of Short-Chain Fatty Acids with a Focus on Propionate

### 4.1. Absorption, Systemic Distribution, and Central Nervous System Accessibility of Propionate

To evaluate whether propionate may be involved in neuro-pathophysiological processes relevant to ASD, it is necessary to consider whether biologically plausible routes exist by which this metabolite can reach the CNS. Propionate produced by gut microbiota is primarily absorbed across the colonic epithelium, a process mediated by both passive diffusion and monocarboxylate transporters.

Following absorption, propionate enters the portal circulation and is delivered to the liver, where a substantial fraction undergoes first-pass metabolism. Hepatic processing includes conversion to propionyl-CoA and subsequent utilization in energy production, gluconeogenesis, or lipid-related metabolic pathways, thereby limiting the proportion of propionate that ultimately reaches the systemic circulation. Consequently, circulating propionate concentrations reflect not only microbial production within the gut, but also colonic epithelial absorption efficiency, intestinal transit time, hepatic metabolic capacity for propionyl-CoA processing, and host metabolic status, including dietary fiber and carbohydrate intake patterns, insulin resistance, and adiposity [94,112]. These considerations indicate that blood propionate levels should be interpreted as an integrated outcome of the gut–liver–systemic metabolic axis rather than as a direct surrogate marker of gut microbial activity alone.

A critical issue in considering potential CNS effects of propionate is its capacity to cross the BBB. Certain SCFAs have been reported to cross the BBB to a limited extent via specific transport mechanisms, and propionate has been proposed, based on evidence from experimental systems, as one such candidate. However, BBB accessibility is likely to be strongly context-dependent, influenced by circulating concentration, exposure duration, transporter expression, and developmental stage [38,39]. During early developmental periods, these properties may differ substantially from adulthood, leading to variability in central exposure and effects across the lifespan [122,123,124,125].

Accordingly, the potential impact of propionate on the CNS should not be inferred solely from its theoretical ability to cross the BBB. Rather, it should be interpreted within a context-dependent framework incorporating developmental stage, exposure conditions, and metabolic environment. Collectively, these considerations suggest that the neurobiological relevance of propionate is shaped by developmental timing and biological context, with key developmental windows summarized in Table 4.

### 4.2. Potential Disruption of the Neurodevelopmental Milieu: Neuronal Differentiation, Synaptogenesis, and Neuroinflammation

When considering the potential impact of propionate on neurodevelopmental processes, current evidence remains insufficient to establish a definitive causal relationship. Most findings are derived from animal or in vitro models, and should therefore be interpreted within the limitations of experimental paradigms, and may not directly reflect human neurodevelopmental processes. Accordingly, this section focuses on the possibility that propionate may influence the neurodevelopmental milieu rather than directly disrupt it.

During brain development, neuronal differentiation and maturation are tightly regulated by metabolic state, immune signaling, and the extracellular microenvironment [126,127]. These regulatory processes are, in turn, influenced by systemic metabolic conditions, including signals derived from gut microbiota metabolism [60,128]. For instance, Yang et al. (2020) reported that physiological, micromolar concentrations of SCFAs (acetate, propionate, and butyrate) significantly increased proliferation rates and the proportion of mitotic cells in human neural progenitor cultures [129]. Complementing these findings, Erny et al. (2015) demonstrated that germ-free mice exhibit immature microglial phenotypes characterized by increased dendritic length, branching points, and terminal processes, suggesting that gut microbiota-derived signals contribute to shaping the immune environment of the developing brain [130].

Beyond postnatal development, evidence has also emerged for a maternal gut–embryonic brain axis through which microbiota-derived metabolites, including SCFAs, may influence embryonic neuronal differentiation and subsequent offspring behavior [131]. In line with this concept, offspring of dams exposed to high-fat diets displayed impairments in cognitive and social behaviors, reduced expression of synaptic proteins such as PSD-95, and delayed microglial maturation. Notably, these alterations were partially reversed by high-fiber dietary interventions or SCFA supplementation during gestation or early postnatal life [132]. Collectively, these findings provide quantitative support for the notion that gut microbiota-derived SCFAs may modulate metabolic, immune, and synaptic regulatory axes within the developing brain, thereby establishing a framework for examining the potential role of individual SCFAs, including propionate.

Within this context, the possible influence of propionate on the neurodevelopmental milieu may involve alterations in metabolic and immune signaling pathways. Several preclinical studies have reported changes in neuronal differentiation markers, synaptic proteins, and glial activation following propionate exposure. However, these findings are largely derived from high-dose or non-physiological experimental conditions and should be interpreted as model-dependent observations rather than direct evidence of neurodevelopmental reprogramming [1,120,129]. As such, these findings are more appropriately interpreted as indications that metabolic–immune regulatory axes can be perturbed under restricted conditions, rather than as conclusive evidence that propionate directly reprograms neurodevelopmental trajectories.

Synapse formation and remodeling warrant similar caution in interpretation. Under normal development, these processes are tightly regulated by neuron-glia interactions and are sensitive to local metabolic and inflammatory states [133,134]. Reported alterations following propionate exposure should therefore be considered context-dependent observations rather than definitive evidence of direct synaptic disruption [135,136].

Neuroinflammatory signaling represents another potential interface linking propionate exposure to changes in the neurodevelopmental environment. Under certain conditions, propionate has been associated with modulation of immune responses and altered activation states of glial cells. Given that microglial activation during development plays a central role in synaptic pruning and circuit maturation, such changes may indirectly influence neurodevelopmental outcomes [120,133,137]. Importantly, however, these associations should be interpreted as contributory rather than causal, reflecting one of multiple regulatory inputs shaping the developing brain.

Crucially, these effects are highly dependent on exposure timing, concentration, duration, and developmental stage. Rather than acting as an acute neurotoxic agent, propionate is more plausibly conceptualized as a context-dependent modulator that may influence neurodevelopmental processes under specific conditions.

### 4.3. Mitochondrial Dysfunction and Energy Metabolic Stress

One of the reported biological features in ASD is vulnerability in mitochondrial function and energy metabolism. These features are generally interpreted as the integrated outcome of multiple biological processes rather than a direct consequence of a single molecular factor. Accordingly, propionate should be considered within this broader metabolic framework.

Mitochondria play an important role in neuronal energy supply and the maintenance of synaptic function, with particularly high energetic demands during neurodevelopment. Several preclinical studies have described changes in mitochondrial indices following propionate exposure, including altered electron transport chain efficiency, dysregulation of mitochondrial membrane potential, and shifts in cellular redox balance. For example, rodent models receiving intracerebroventricular propionate administration have demonstrated reduced activity across multiple respiratory chain complexes, decreased ATP production, and increased reactive oxygen species (ROS) generation under experimental conditions. Similarly, cell-based studies using lines derived from individuals with ASD and from controls have shown that propionate exposure is associated with alterations in oxygen consumption rates, mitochondrial membrane potential, ROS production, and ATP synthesis, suggesting modulation of mitochondrial electron transport and redox homeostasis under certain conditions. In addition, tissues from patients with propionic aciduria—a metabolic disorder characterized by chronic propionate accumulation—have demonstrated secondary mitochondrial abnormalities, including reduced respiratory chain complex activity, decreased mitochondrial DNA content, and structural mitochondrial alterations [138,139,140].

Importantly, much of the evidence linking propionate exposure to mitochondrial dysfunction is derived from experimental models, including animal and in vitro systems. In addition, some mechanistic insights are informed by studies of propionic aciduria, which is etiologically distinct from ASD. Accordingly, these findings do not establish a direct causal relationship between propionate and mitochondrial dysfunction in individuals with ASD, and extrapolation to the broader ASD population should therefore be approached with caution.

From an energy metabolism perspective, multiple studies have reported abnormalities in TCA cycle intermediates and pyruvate-related metabolites in urine, blood, and brain tissue from individuals with ASD [141,142]. Elevated lactate concentrations and increased lactate-to-pyruvate ratios in peripheral blood have been observed across several cohorts [143,144], commonly interpreted as indirect indicators of impaired mitochondrial respiratory efficiency or altered cytosolic–mitochondrial redox balance. In some cohorts, reduced activity of electron transport chain complex I and concomitant decreases in ATP-generating capacity have been detected in peripheral blood cells from children with ASD [145]. Consistent patterns have also been reported in stem cell-based models derived from individuals with ASD, which demonstrated slowed glycolytic flux, ATP deficiency, and reduced cellular respiration [146]. Together, these observations suggest the presence of an energy metabolic stress state that may impose additional constraints on neuronal function.

In parallel with these observations, several studies in individuals with ASD have identified alterations in mitochondrial function independent of propionic aciduria. For example, Giulivi et al. (2010) identified a subset of children with ASD exhibiting mitochondrial dysfunction, including reduced activity of electron transport chain complexes and increased oxidative stress markers [145]. Additional reports have described alterations in mitochondrial respiration, ATP production, and redox balance in peripheral tissues and ASD-derived cellular models [145,146]. Importantly, these findings are not consistently observed across all cohorts and instead reflect substantial heterogeneity within the ASD population.

Propionate is metabolized within host cells through conversion to propionyl-CoA and methylmalonyl-CoA, ultimately yielding succinyl-CoA and allowing entry into the TCA cycle. Experimental studies in animal models and hepatocyte systems have suggested that propionate exposure can increase TCA cycle intermediate pools and enhance pyruvate cycling, indicative of elevated anaplerotic flux. Under certain conditions, such metabolic loading may place additional strain on energy homeostasis [69,147]. The lactate-to-pyruvate ratio, which reflects cytosolic NADH/NAD^+^ redox status via the lactate dehydrogenase reaction, has repeatedly been reported to increase in contexts of mitochondrial dysfunction and metabolic stress, including ASD [143,148].

Although several preclinical studies have reported changes following propionate exposure, these findings do not support a uniform causal model linking propionate to mitochondrial dysfunction in ASD. Rather, they suggest that propionate may act as a context-dependent modulator that may amplify existing metabolic imbalance under conditions of vulnerability [138,139]. This interpretation aligns with a framework in which propionate is viewed not as a singular etiological agent, but as a context-dependent stress amplifier acting upon an already susceptible metabolic background.

**Figure 1:**
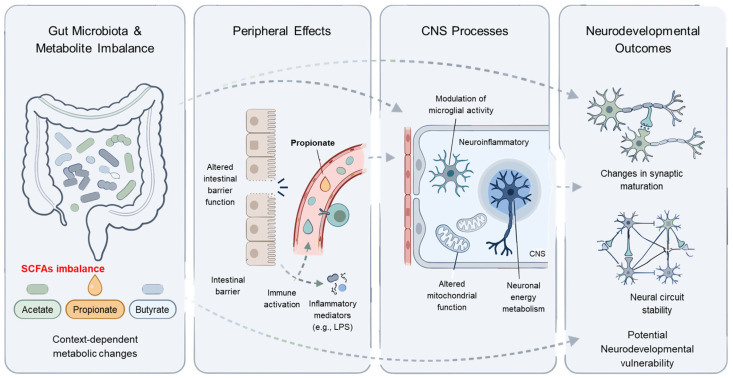
Conceptual summary of proposed mechanisms linking gut-derived propionate to neurobiological processes in autism spectrum disorder. This schematic illustrates a conceptual and hypothesis-generating overview of how gut-derived propionate may be associated with neurobiological processes relevant to ASD. The relationships depicted represent proposed and context-dependent associations rather than direct causal pathways. Alterations in gut microbiota composition may be associated with imbalances in short-chain fatty acids (SCFAs), including acetate, propionate, and butyrate, reflecting context-dependent metabolic conditions. Under certain biological conditions, these changes may be accompanied by alterations in intestinal barrier function and immune activation, potentially facilitating the systemic circulation of microbial metabolites (e.g., propionate) and inflammatory mediators such as lipopolysaccharide (LPS). These peripheral signals may be conveyed to the central nervous system through multiple interacting pathways. Within the CNS, such signals have been reported to be associated with modulation of microglial activity, neuroinflammatory tone, mitochondrial function, and neuronal energy metabolism under specific experimental and biological contexts. These processes may, in turn, be associated with changes in synaptic maturation and neural circuit stability, potentially contributing to context-dependent neurodevelopmental vulnerability. Importantly, the mechanisms depicted are primarily derived from preclinical and model-based evidence, and should therefore be interpreted with caution in terms of their translational relevance to human ASD. The arrows represent the proposed directional pathways and interactions between gut-derived metabolites and neurobiological processes.

Oxidative stress represents an additional dimension closely linked to mitochondrial dysfunction. While direct causal evidence remains limited, increased metabolic stress may contribute to secondary oxidative burden under certain conditions [144,145]. To aid interpretation of the complex and context-dependent mechanisms discussed above, a schematic summary of the proposed processes, with a particular focus on propionate, is provided in Figure 1.

### 4.4. Distinguishing Direct Effects from Axis-Mediated Indirect Effects

The neuro-pathophysiological implications of propionate may be broadly categorized into direct effects and axis-mediated indirect effects. These pathways should be considered complementary rather than mutually exclusive and may operate concurrently. Accordingly, the relevance of propionate is better interpreted as a context-dependent signal reflecting the functional state of the broader gut–immune–metabolic–brain axis, rather than as an isolated causal factor.

## 5. Retrograde Signaling via the Gut–Brain Axis

### 5.1. Conceptual Framework of Gut Metabolite-Based Bottom-Up Signaling

The gut–brain axis has traditionally been understood as a bidirectional communication system linking the GI tract and the CNS. More recently, increasing attention has shifted toward bottom-up (periphery-to-brain) signaling, whereby alterations in the intestinal environment may influence CNS function [149,150]. From this perspective, gut microbiota and their derived metabolites can be viewed as signaling mediators that translate local intestinal changes into neural regulatory effects.

Gut metabolite-based retrograde signaling is best conceptualized as a multiaxial system integrating neural, immune, and metabolic pathways. Neural signaling may occur through vagal afferents responsive to luminal metabolites, while impaired intestinal barrier integrity can permit translocation of microbial components, triggering systemic immune activation, which has been associated, primarily in experimental studies, with activation of central immune signaling pathways and increased expression of inflammatory mediators such as TNF-α and IL-6. These parallel pathways reflect complementary routes through which peripheral signals may influence CNS function [151,152,153].

Together, these pathways indicate that the gut–brain axis can be conceptualized as an integrated regulatory system linking peripheral physiological states to central responses. Within this framework, metabolites such as propionate are more appropriately interpreted as context-dependent signaling outputs rather than isolated pathogenic agents.

### 5.2. Signal Amplification Mediated by Intestinal Barrier Dysfunction and Systemic Inflammation

Within the framework of retrograde signaling, intestinal barrier integrity represents a critical determinant of whether gut-derived signals can influence systemic and central processes. Under physiological conditions, tight junction proteins maintain epithelial integrity, whereas factors such as dysbiosis, inflammation, and metabolic stress can disrupt this barrier [154,155,156]. In support of this mechanism, Crawford et al. (2022) reported, using intestinal organoid models, that exposure to TNF-α or IFN-γ induces structural distortion of tight junctions, characterized by increased tortuosity (*p* < 0.0001), along with enhanced epithelial permeability and dysregulated epithelial cell cycle control, providing direct evidence that inflammatory cytokines can impair barrier integrity [157].

When epithelial barrier function is compromised, microbial-derived molecules and gut metabolites may translocate into the systemic circulation. Previous studies have shown that barrier disruption facilitates LPS translocation and systemic immune activation, with evidence from both human-related and animal models demonstrating movement of microbial components beyond the gut environment [158,159].

Such translocation can occur in the absence of overt infection and may contribute to chronic low-grade inflammation, characterized by elevated circulating inflammatory mediators [160]. This inflammatory state may further reinforce barrier dysfunction, creating a feedback loop that may amplify peripheral and systemic signaling [161]. Within this inflammatory milieu, gut-derived metabolites function as components of a composite signaling environment rather than acting in isolation.

Systemic inflammation has particular relevance for CNS involvement. In a mouse model, Huo et al. (2021) reported that LPS translocation secondary to intestinal barrier disruption was associated with activation of TLR4/MyD88 signaling in brain tissue, leading to increased expression of microglial activation markers (Iba-1 and CD68) and elevated levels of pro-inflammatory cytokines including TNF-α and IL-6 [153]. Notably, these changes were attributed not to direct neurotoxicity of gut-derived metabolites, but to the combined effects of epithelial barrier dysfunction and systemic inflammatory activation.

SCFAs, including propionate, may exert different effects depending on barrier integrity. Under intact conditions, they may support barrier function and immune regulation, whereas compromised barrier states may alter systemic exposure and downstream signaling effects [34,43]. This highlights the context-dependent nature of SCFA functions within gut–brain communication.

### 5.3. Integration of Neuro–Immune–Metabolic Pathways and Central Nervous System Responses

The downstream consequences of retrograde signaling are unlikely to converge on a single response, but rather reflect integration of neuro–immune–metabolic pathways [161,162].

Microglia play an important role in linking immune signaling to neural circuit regulation. Experimental studies have suggested that peripheral immune activation can induce sustained microglial activation and neuroinflammatory responses, including prolonged cytokine elevation and neuronal vulnerability [133,163,164]. However, these findings are derived from specific experimental conditions and should be interpreted with caution in relation to human ASD.

From a metabolic perspective, systemic inflammation may impair neuronal energy metabolism, reducing ATP availability and compromising synaptic function [165,166]. Given that synaptic transmission accounts for a substantial proportion of cerebral energy consumption, even modest metabolic disturbances may impose significant constraints on neuronal function.

Crucially, neuro–immune–metabolic responses do not operate in isolation but tend to form self-amplifying interaction networks. Immune activation can drive a metabolic shift in microglia toward glycolysis-dominant states, and this metabolic reprogramming, in turn, enhances cytokine production and release, further amplifying immune signaling. Such positive feedback loops suggest that retrograde signaling may be fundamentally state-dependent, with coordinated regulatory changes persisting even after the initial peripheral stimulus has subsided. This interactional architecture provides a mechanistic basis for sustained neuro–immune–metabolic coupling following transient peripheral perturbations [167,168].

### 5.4. Limitations of Preclinical Evidence and Clinical Implications

Although the concept of retrograde signaling is primarily supported by preclinical studies, significant limitations remain regarding reproducibility and interpretation. Many findings are derived from non-physiological or model-specific conditions, leading to variability across studies [123,131,135]. Clinical studies likewise show inconsistent findings, with associations between SCFAs and ASD varying across cohorts and methodologies [47,48]. Such discrepancies highlight the influence of study design and analytical approaches on observed outcomes.

More fundamentally, it is important to recognize the structural limitations inherent to preclinical approaches that rely on microbiota transplantation, experimental disruption of intestinal barrier integrity, or artificial induction of specific metabolic states. While these strategies are valuable for exploring conceptual plausibility, they often simultaneously influence gut ecology, immune responses, and metabolic conditions, making it difficult to isolate the independent effects of a single metabolite [46,114]. Consequently, observations derived from such models cannot be readily attributed solely to direct causal effects of individual metabolites. Instead, preclinical evidence supporting retrograde signaling should be interpreted as exploratory indications of potential mechanistic pathways rather than as definitive evidence of their operation in human ASD pathophysiology.

An additional issue concerns dose relevance. In many experimental studies, the concentrations of propionate used exceed those typically observed in human physiological conditions. While such approaches can be useful for probing underlying mechanisms, they limit the extent to which these findings can be directly translated to human ASD. Accordingly, caution is needed when interpreting these results in a real-world biological context.

It is also important to consider the possibility of publication bias. The field of microbiota–gut–brain axis research in ASD has grown rapidly, and studies reporting positive or statistically significant findings may be more likely to be published. This tendency can lead to an imbalance in the literature, with supportive evidence being more visible than negative or inconclusive results. As a result, the overall strength and consistency of reported associations may be overstated.

A related issue is reverse causality. Changes in gut microbiota observed in individuals with ASD do not necessarily indicate a primary causal role. In some cases, these alterations may arise as a consequence of behavioral features of ASD, such as selective eating or restricted dietary patterns. This makes it difficult to disentangle cause and effect and suggests that microbial changes should not be interpreted as inherently causal without further supporting evidence.

Taken together, these considerations support a cautious and context-dependent interpretation of retrograde signaling frameworks. Rather than representing direct causal pathways, these mechanisms are better understood as integrated, context-sensitive processes linking gut physiology to central nervous system function. Importantly, these processes are unlikely to operate uniformly across all individuals with ASD, and their relevance may depend on specific biological and clinical contexts. This perspective provides a more balanced foundation for interpreting the proposed “metabolic ASD” framework.

## 6. Gut-Derived Metabolites and ASD: Integration of Clinical Evidence, Boundaries of Interpretation, and a Research Roadmap for Precision Interventions

### 6.1. Integrative Interpretation of Clinical Evidence: Signals Are Present, but Not Universal Markers

As summarized in Section 3, numerous studies have examined gut microbiota-related metabolites, particularly SCFAs, in ASD cohorts [40,47]. However, reported findings vary substantially across studies and do not support consistent alterations in specific SCFAs at the population level [47,48,97,98]. Accordingly, the key implication lies not in the presence or absence of SCFA changes, but in identifying the clinical and biological contexts in which these metabolic signals become salient.

This context dependence is clearly reflected in sampling location. Fecal SCFAs primarily reflect luminal concentrations within the gut, whereas circulating SCFAs represent integrated outcomes of absorption, hepatic metabolism, and systemic utilization [112,113]. Thus, discrepancies across studies may reflect differences in physiological layers rather than methodological inconsistency.

Importantly, several studies report more pronounced metabolic alterations in individuals with ASD presenting with co-occurring GI symptoms, suggesting selective rather than universal relevance [97,98,111]. Taken together, current evidence supports a context-dependent interpretation of gut-derived metabolic signals within ASD heterogeneity. This perspective highlights the importance of stratified approaches integrating clinical and biological features (Table 5).

### 6.2. Guarding Against Premature Causal Inference: Reframing “Limitations” as Design Requirements

The recurrent inconsistencies observed in studies of gut-derived metabolites should not be interpreted as insufficient evidence, but rather as an indication of the need for specific study designs to enable clinically meaningful interpretation.

First, evaluation of causality and temporal precedence highlights the need to move beyond cross-sectional analyses toward developmentally informed longitudinal designs. ASD is fundamentally characterized by altered developmental trajectories, and both gut microbiota composition and metabolic environments change dynamically with age, diet, and living conditions [149]. Accordingly, longitudinal tracking of the same individuals is important for determining whether changes in metabolic markers precede, follow, or co-evolve with alterations in clinical phenotypes.

Second, study designs should incorporate stratification that reflects the clinical heterogeneity of ASD. At a minimum, subgrouping based on the presence of GI symptoms, age range, dietary restrictions or selective eating patterns, comorbid metabolic states (e.g., obesity or insulin resistance), and histories of medication or antibiotic exposure should be considered important design considerations rather than optional adjustments. Without such stratification, comparisons based on group-level averages risk conflating biologically distinct conditions and obscuring meaningful signal patterns [100,108,109,110].

Third, ensuring reproducibility in gut metabolite research requires standardized pipelines for sample processing, analysis, and reporting. Variability in storage conditions, extraction methods, internal standards, analytical platforms (e.g., GC–FID, GC–MS, LC–MS/MS), and reporting formats (absolute concentrations versus relative proportions) can result in divergent quantitative outcomes even when similar biological phenomena are examined [115,118]. At a minimum, consensus on core SCFA panels and reporting units may be needed to facilitate cross-study comparability.

Fourth, interpretation of SCFA-related findings should move beyond isolated metabolite measurements toward multilayered biomarker frameworks that capture intestinal barrier integrity, systemic inflammation, and metabolic state. As discussed in Section 5, gut–brain axis-mediated effects are rarely reducible to the direct action of a single metabolite; rather, they are more appropriately understood as state changes arising from the convergence of epithelial barrier dysfunction, inflammatory activation, and metabolic stress [149,158,161]. Concurrent assessment of barrier-related markers (e.g., zonulin), LPS-associated indices, inflammatory cytokines, and indicators of mitochondrial function or oxidative stress would allow metabolic signals to be interpreted within their physiological context.

Ultimately, this section aims not to emphasize limitations, but to define the minimum design criteria required to establish “metabolic ASD” as a testable framework. Future progress will likely depend on the extent to which these design requirements are systematically implemented.

For example, this framework can be operationalized through longitudinal cohort designs that stratify individuals based on metabolic and GI profiles and examine their association with neurodevelopmental trajectories. Such approaches enable the formulation of testable hypotheses regarding whether specific metabolic profiles precede or modify ASD-related phenotypes.

### 6.3. Therapeutic Implications of the Metabolic ASD Concept: Defining the Target and the Endpoints, Not the Magnitude of Effect

As interest in gut-derived metabolic imbalance in ASD has increased, the limitations of treating ASD as a homogeneous condition have become increasingly evident. Accordingly, therapeutic strategies may benefit from moving beyond population-level efficacy toward more individualized and biologically informed approaches that account for heterogeneity across individuals.

To date, proposed therapeutic strategies can be broadly categorized into three approaches:

(i) Modulation of gut microbial ecology through probiotics or prebiotics [11,169];
(ii) Dietary interventions such as fiber enrichment or targeted carbohydrate modulation [170];
(iii) Microbiota reconstitution approaches, including fecal microbiota transplantation [149].

These approaches should be understood within a broader, multifactorial framework, where gut-derived metabolic modulation represents one of several interacting dimensions rather than an isolated therapeutic target.

Among dietary approaches, microbiota-modulating dietary patterns such as the Mediterranean diet have been reported to influence gut microbial composition and metabolic outputs, including SCFAs. These effects have been associated with broader impacts on host metabolic and inflammatory states. However, current evidence does not support a consistent or direct association with ASD-specific clinical outcomes, and their effects are likely to be context-dependent [171].

Although some studies have reported changes in gut microbiota composition or metabolic indices following these interventions, effects on core ASD symptoms have not been consistently replicated at the population level. When clinical improvements have been observed, they have typically appeared in selected subgroups rather than across entire cohort [47,48,169,172]. These observations underscore that the central challenge is not to overstate the promise of specific interventions, but to refine the criteria for identifying responders and define biologically meaningful endpoints.

At least three methodological steps may be necessary to advance this approach. First, biomarker-informed stratification should precede intervention. Second, treatment evaluation should include mechanism-linked endpoints beyond behavioral outcomes. Third, longitudinal and translational designs are needed to clarify temporal relationships between metabolic changes and clinical phenotypes.

**Figure 2:**
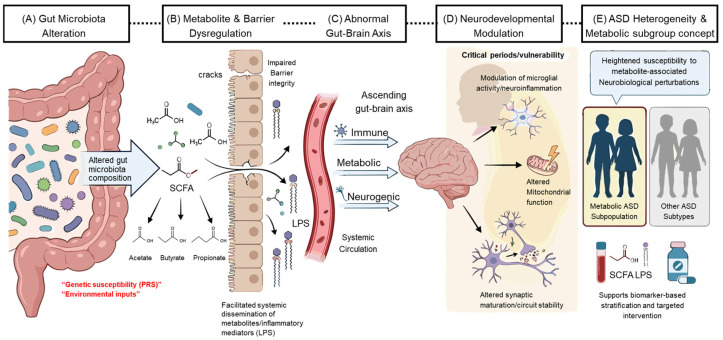
Conceptual framework of a metabolically defined subtype of autism spectrum disorder associated with gut-derived metabolite imbalance. This figure presents a conceptual and hypothesis-generating model proposing the existence of a metabolically defined subtype of ASD, in which gut-derived metabolite imbalance acts as a context-dependent pathophysiological contributor rather than a universal etiological factor. The relationships illustrated are intended to represent proposed and context-dependent associations rather than direct causal pathways. (**A**) Alterations in gut microbiota composition may be associated with quantitative and qualitative imbalances in short-chain fatty acids (SCFAs), including acetate, propionate, and butyrate. (**B**) Under biologically susceptible conditions, dysbiosis-associated metabolic disturbances may contribute to impaired intestinal epithelial barrier integrity, potentially facilitating the systemic dissemination of microbial metabolites and inflammatory mediators such as lipopolysaccharide (LPS). (**C**) These peripheral signals may be conveyed to the CNS through ascending gut–brain axis pathways, encompassing immune, metabolic, and neurogenic routes. (**D**) During critical neurodevelopmental periods, such signals may influence microglial activity, neuroinflammatory tone, mitochondrial function, and neuronal energy metabolism, and may be associated with changes in synaptic maturation and neural circuit stability. (**E**) This model highlights ASD heterogeneity by delineating a “metabolic ASD” subpopulation characterized by heightened susceptibility to metabolite-associated neurobiological perturbations, providing a conceptual basis for biomarker-guided stratification. This schematic also illustrates how genetic susceptibility (e.g., polygenic risk) and environmental factors may interact with gut-related metabolic and immune pathways, providing a conceptual framework for testable hypotheses consistent with current gene–environment interaction models of ASD.

In summary, therapeutic approaches based on the metabolic ASD framework should be viewed as precision strategies applicable to specific subgroups rather than universal interventions. An important priority is to establish robust stratification and study designs that enable biologically meaningful and testable therapeutic hypotheses, as summarized in Figure 2, which integrates genetic susceptibility, environmental influences, and peripheral metabolic processes into a unified framework of ASD heterogeneity.

## 7. Conclusions

ASD is a highly heterogeneous neurodevelopmental condition that cannot be adequately explained by a single pathophysiological mechanism. This heterogeneity underscore the need for integrative conceptual frameworks that account for interactions among genetic, environmental, and biological factors. In this review, we examined gut microbiota-derived metabolic imbalance, particularly SCFAs, and introduced the concept of “metabolic ASD” as a hypothesis-generating framework to capture one potential dimension of biological variability, rather than a discrete or mutually exclusive subtype. Within this framework, peripheral metabolic and immune environments are considered interacting components that may contribute to neurodevelopmental vulnerability in a context-dependent manner, particularly during periods of heightened sensitivity to metabolic and immune signals. Accordingly, gut-derived metabolic imbalance is more appropriately interpreted as a phenotypic modifier rather than a causal determinant or diagnostic biomarker.

Across clinical studies, reported alterations in SCFA concentrations exhibit substantial heterogeneity and strong context dependence. Rather than representing a universal feature of ASD, SCFA imbalance reflects signals shaped by measurement conditions, developmental stage, and clinical characteristics. These findings underscore the limitations of generalizing ASD pathophysiology based on single metabolites or isolated pathways.

Within this heterogeneous landscape, propionate has been frequently investigated due to its associations with neuroimmune, metabolic, and behavioral processes. However, current evidence does not support a direct etiological role. Instead, propionate is more appropriately interpreted as a context-dependent signal whose effects may be amplified under specific metabolic and immune conditions.

The integrative perspective presented in this review links gut microbiota-derived metabolic signals to CNS function through barrier integrity, systemic inflammation, and neuro–immune–metabolic interactions. This framework is intended to guide interpretation of ASD heterogeneity and support the development of testable, subgroup-specific research models, rather than to establish causality or diagnostic criteria.

In summary, ASD cannot be reduced to a single biological axis. In a subset of individuals, a metabolic axis centered on gut-derived metabolites may contribute to neurodevelopmental susceptibility. The concept of metabolic ASD should therefore be viewed as a hypothesis-generating framework for refining study design, enabling biologically informed stratification, and guiding precision-oriented interventions. Its clinical relevance will likely require validation through longitudinal and subgroup-focused studies and should be incorporated into research design rather than applied as a standalone diagnostic classification.

## Figures and Tables

**Table 1:** Reported alterations in short-chain fatty acid levels in autism spectrum disorder across human studies.

Study	Model	Sample Type	Age	Number of Patients (*n*)		ASD Characteristics	Analysis Method	ASD Patients		
				ASD	Cont			Acetate	Propionate	Butyrate
Wang et al., 2019 [47]	Human	Feces	Children	45	90	ASD vs. TD controls	HPLC	↔	↔	↔
Liu et al., 2019 [48]	Human	Feces	Children	30	20	ASD vs. TD controls	HPLC	↓	NS	↓
He et al., 2023 [97]	Human	Feces	Children	40	40	ASD with constipation vs. TD controls	GC-MS	↑	↔	↔
Deng et al., 2022 [98]	Human	Feces	Children	45	45	ASD vs. TD controls	GC-MS	↑	↑	↑

Table legend. Summary of reported alterations in SCFA levels in ASD based on human case–control studies that performed direct quantitative comparisons with control groups. Arrows indicate the direction of statistically significant changes relative to controls (↑ increased, ↓ decreased, ↔ no significant change). Differences across studies reflect variation in biological and methodological context, including sample source, gastrointestinal symptom stratification, developmental stage, and analytical methodology. SCFAs, short-chain fatty acids; ASD, autism spectrum disorder; Cont, Control; GI, gastrointestinal; TD, typically developing.

**Table 2:** Dominant gut microbial taxa reported as altered in autism spectrum disorder across human cohort studies.

Study	Model	Sample Type	Increased Taxa	Decreased Taxa	Functional Implication
Liu et al., 2019 [48]	Human (ASD)	Feces	Propionate- and acetate-producing taxa; *Desulfovibrio* (G)	Butyrate-associated taxa; Ruminococcaceae (F), *Eubacterium* (G), Lachnospiraceae (F), Erysipelotrichaceae (F)	Altered SCFA-related microbial balance
Li et al., 2024 [99]	Human (ASD)		Fusobacteria (P), Firmicutes (P), Verrucomicrobia (P)	Lentisphaerae (P), Bacteroidetes (P), Euryarchaeota (P), Patescibacteria (P)	Altered microbial community structure
Lou et al., 2022 [100]	Human (ASD)		*Bifidobacterium* (G), *Veillonella* (G), Enterobacteriaceae (F), Lachnospiraceae (F)	*Clostridium* (G), *Veillonella ratti* (S)	Early-life cohort-specific microbial deviation
Iglesias-Vázquez et al., 2020 [102]	Human (ASD)		*Bacteroides* (G), *Parabacteroides* (G), *Clostridium* (G), *Faecalibacterium* (G), *Phascolarctobacterium* (G)	*Coprococcus* (G), *Bifidobacterium* (G)	Context-dependent lack of a consistent ASD microbial signature
Kang et al., 2013 [103]	Human (ASD)		NR	*Prevotella* (G), *Coprococcus* (G), *unclassified* Veillonellaceae (F)	Disrupted microbial interactions
Coretti et al., 2018 [104]	Human (ASD)		Bacteroidetes (P), Proteobacteria (P)	Actinobacteria (P)	Pro-inflammatory microbial shift

Table legend. Summary of dominant gut microbial taxa reported to be altered in individuals with ASD across representative human cohort studies. Taxa are listed at the genus or higher taxonomic level when consistently reported, as taxonomic resolution varies across study designs and analytical pipelines. This comparison highlights both convergent and divergent microbial patterns across ASD cohorts, underscoring the context-dependent and non-universal nature of gut microbiota alterations. The table also illustrates the limitations of direct taxonomic translation across studies, emphasizing that reported microbial shifts should be interpreted within their specific clinical, developmental, and methodological contexts. ASD, autism spectrum disorder; SCFAs, short-chain fatty acids.

**Table 3:** Major sources of biological and methodological heterogeneity influencing reported SCFA alterations in autism spectrum disorder studies.

Source of Heterogeneity	Categories/Examples	Impact on SCFA Readouts	Implications for Interpretation
Sample source	Feces vs. plasma/serum	Captures luminal fermentation output vs. absorbed/systemically available SCFAs, influenced by host metabolism (e.g., hepatic clearance)	Fecal and circulating SCFAs are not interchangeable
GI symptom stratification	Constipation, diarrhea, abdominal pain	Alters intestinal transit time, fermentation kinetics, absorption efficiency, and stool water content	Null findings may reflect signal dilution in unstratified cohorts
Developmental stage	Infancy, childhood, adolescence	Age-dependent microbiota maturation and diet transitions shift baseline SCFA profiles	Age mismatch limits cross-study comparability
Diet and fiber intake	High vs. low fermentable fiber; dietary patterns	Directly modulates microbial substrate availability and SCFA production	Dietary control is critical for stronger causal inference
Medication exposure	Antibiotics, probiotics/prebiotics, laxatives, psychotropics, PPIs	Reshapes microbial composition and metabolic output; may interact with GI status	Medication history should be systematically captured and adjusted for
Analytical workflow/platform	GC-FID, GC–MS, LC–MS/MS (with derivatization and internal standards)	Differences in sensitivity, specificity, and quantification range across platforms	Methodological heterogeneity contributes substantially to variability
Reporting units/normalization	Absolute concentration vs. relative proportion; wet vs. dry weight; different unit scales	Limits quantitative comparability and may invert apparent group differences	Directional interpretation is frequently more appropriate than magnitude alone
Experimental design/model	Human cohorts vs. rodent models; exposure-based vs. endogenous metabolism	Exposure paradigms may not recapitulate endogenous SCFA dynamics in humans	Animal findings should not be directly generalized to human ASD

Table legend. Major sources of biological and methodological heterogeneity contributing to variability in reported SCFA findings in ASD studies. This table summarizes key contextual factors spanning sample source, developmental timing, gastrointestinal comorbidity, diet and medication exposures, analytical workflows, reporting units, and experimental design. Collectively, these sources of variability support interpreting SCFAs as context-dependent metabolic signals rather than universal biomarkers of ASD. SCFAs, short-chain fatty acids; ASD, autism spectrum disorder.

**Table 4:** Developmental window-dependent contexts linking gut-derived short-chain fatty acids to neurodevelopment.

Developmental Stage	SCFA-Related Context	Biological Processes Potentially Affected	Observed or Reported Outcomes	Representative References
Pregnancy (maternal)	Circulating maternal SCFAs reflecting gut fermentation	Maternal metabolic–immune milieu influencing fetal neurodevelopmental susceptibility	Associations with offspring neurodevelopmental measures in cohort studies	[93]
Prenatal period (maternal immune activation)	SCFAs–immune crosstalk potentially intersecting with maternal inflammation	Immune signaling at the maternal–fetal interface	Increased vulnerability to neurodevelopmental alterations in offspring	[14,95]
Early postnatal (infancy)	Infant gut fermentation-derived SCFAs with limited systemic availability	Microglial maturation and synaptic pruning (preclinical support)	Atypical early developmental trajectories in early-life microbiome studies	[90,92]
Childhood	Diet-modulated gut and plasma metabolomic profiles	Systemic metabolic shifts linked to microbiota modulation	Context-dependent associations with autism-related traits	[108,114]
Juvenile (experimental animal models)	Experimental SCFA exposure (e.g., propionate/PPA)	Neuroinflammation and mitochondrial stress pathways	ASD-like behavioral phenotypes in specific paradigms	[1,94]

Table legend. Developmental window-dependent contexts in which gut-derived SCFAs have been reported to associate with neurodevelopmental processes. Representative references are provided to anchor key developmental contexts rather than to exhaustively catalog all relevant studies. Descriptions are intentionally framed as context-dependent and non-deterministic, reflecting differences in developmental timing, biological susceptibility, and experimental paradigms. SCFAs, short-chain fatty acids; ASD, autism spectrum disorder; PPA, propionic acid.

**Table 5:** Core biomarker domains for stratifying a metabolically vulnerable subgroup within autism spectrum disorder.

Biomarker Domain	Candidate Markers	Measurement Source	Biological Rationale
Gut-derived metabolites	Relative SCFA patterns (acetate, propionate, butyrate)	Feces/plasma	Reflects microbial fermentation patterns and systemic availability
Gastrointestinal integrity	Lipopolysaccharide-binding protein (LBP)	Serum	Indicates barrier-related metabolite translocation
Immune activation	Representative inflammatory cytokines (e.g., IL-6, IL-17A)	Serum	Captures inflammatory tone linked to metabolic signaling
Oxidative stress/redox imbalance	Redox-related markers	Blood	Reflects oxidative imbalance relevant to neuronal vulnerability
Mitochondrial/energy metabolism	Energy metabolism–related indicators	Blood	Reflects altered cellular energy metabolism
Microbiota structure	Relative depletion of fermentative taxa	Fecal sequencing	Reflects reduced SCFA-producing capacity
Developmental timing	Perinatal/early-life exposures	Clinical history	Identifies sensitive developmental windows

Table legend. Core biomarker domains proposed to support biological stratification of a metabolically vulnerable subgroup within ASD. The listed domains are research-oriented and non-diagnostic, intended to guide hypothesis-driven study design and context-aware interpretation of metabolic–immune alterations rather than clinical classification. Candidate markers and measurement sources are provided as representative examples reflecting convergent evidence across human and experimental studies. These domains should be interpreted as integrative dimensions rather than standalone biomarkers, emphasizing heterogeneity and context dependence across ASD populations. ASD, autism spectrum disorder; SCFA, short-chain fatty acid.

## Data Availability

No new data were created or analyzed in this study.
